# Differential diagnosis of bovine intestinal diseases and their sequelae regarding ultrasonography and other diagnostic tools

**DOI:** 10.14202/vetworld.2021.1537-1547

**Published:** 2021-06-16

**Authors:** Arafat Khalphallah, Hanan K. Elsayed, Enas Elmeligy, Sara A. Bayomi, Mohamed A. Hamed, Doaa Salman, Ashraf M. Abu-Seida, Sabry A. Mousa

**Affiliations:** 1 Division of Internal Medicine, Department of Animal Medicine, Faculty of Veterinary Medicine, Assiut University, Assiut 71526, Egypt; 2 Veterinary Teaching Hospital, Faculty of Veterinary Medicine, Assiut University, Assiut 71526, Egypt; 3 Division of Clinical Laboratory Diagnosis, Department of Animal Medicine, Faculty of Veterinary Medicine, Assiut University, Assiut 71526, Egypt; 4 Department of Surgery, Anesthesiology and Radiology, Faculty of Veterinary Medicine, Aswan University, Aswan 81528, Egypt; 5 Division of Clinical Laboratory Diagnosis, Department of Animal Medicine, Faculty of Veterinary Medicine, Sohag University, Sohag 82524, Egypt; 6 Department of Surgery, Anesthesiology and Radiology, Faculty of Veterinary Medicine, Cairo University, Giza, Egypt; 7 Division of Internal Medicine, Department of Medicine and Infectious Disease, Faculty of Veterinary Medicine, Cairo University, Giza, Egypt

**Keywords:** caecal dilatation, cattle, ileus, intussusception, peritonitis, ultrasonography

## Abstract

**Background and Aim:**

Intestinal disorders represented a large proportion of abdominal emergencies in bovine practice, and their definite diagnosis was a big challenge. The study described different intestinal disorders in cattle either in the small intestine (SI) or large intestine with their sequelae and peritonitis between SI loops.

**Materials and Methods:**

This study involved healthy (n=20) and diseased (n=40) cattle with intestinal disorders. All animals were undergoing clinical examination, laboratory analyses, and ultrasonographic examination.

**Results:**

Diseased cattle had monocytic leukocytosis as well as hypoproteinemia and hypoalbuminemia. The SI ileus of either proximal (n=12) or distal (n=15), intussusception (n=3), cecal, and/or colonal dilatation (n=10) were diagnosed by ultrasound and other diagnostic tools. Duodenum intussusception was imaged in cross-section as bull’s eyes lesions. Animals with SI obstructions showed complete cessation (ileus with complete obstruction) or partial reduction of the peristaltic SI movement (ileus with partial obstruction), dilated duodenum (6.5-9.9 cm), and dilated jejunum and/or ileum (4.4-6.8 cm). Ultrasonography diagnosed SI ileus, due to either intestinal obstruction or peritonitis, and detected the ileus site, either proximal or distal. Cecal/colonal dilatation was detected using ultrasonography in which SI was not imaged and the peristaltic movements were completely reduced. The recorded intestinal disorders were associated with other disorders (e.g., liver cirrhosis or peritonitis).

**Conclusion:**

Ultrasonography played an important role in the differential diagnosis of intestinal disorders in cattle. Peritonitis between SI loops and cecal and/or colonal dilatation was also diagnosed.

## Introduction

Preliminary diagnosis of gastrointestinal disorders in ruminants is achievable by the usual diagnostic measures (e.g., visual inspection, palpation, percussion, and auscultation) [1-3]. However, a definitive diagnosis requires ultrasonography [4-8]. Intestinal obstruction (IO) is a potentially life-threatening disorder in all large animals, particularly in horses [9]. Moreover, it was also reported in cattle [10] and buffaloes [5] and rarely diagnosed in sheep and goats (except for intestinal volvulus in lambs) [11]. Two common IO types occur in large ruminants: mechanical and functional IO [5,11,12]. The etiology of mechanical IO may be luminal or extraluminal. Luminal obstructions (e.g., hemorrhagic jejunitis, trichobezoars, phytobezoars, cecocolic volvulus, impacted ingesta, and atresia coli, recti, and ani) were reported [5,6,13]. Extraluminal obstructions of the gastrointestinal tract (e.g., strangulation, intussusceptions, and volvulus) and intestinal compression with an expanding abdominal mass such as fat necrosis or lymphosarcoma also occurred [14,15].

In contrast to a mechanical obstruction, functional obstruction (paralytic ileus) occurred due to the cessation of peristaltic movement of the intestinal tract. The exact cause of functional stenosis was not well-known but associated with management or dietary factors, parasite infection, phytobezoars, enteritis, peritonitis, or electrolyte disturbances [11]. Paralytic ileus had no gross abnormality but was characterized by reduced intestinal motility or atony. This condition occurred more frequently than mechanical obstruction, particularly in pregnant and recently parturient cows [11,16]. Animals with paralytic ileus showed unspecific clinical and rectal findings [12]. Therefore, another diagnostic tool, like ultrasonography, was recommended to confirm the final diagnosis.

Cecal dilatation is common in dairy cows during the first few months of lactation. The cecum may be distended with gases or ingesta, and volvulus may occur. Loss of appetite, drop in milk yield, reduced feces amount, abdominal discomfort, ping sound over the right upper flank, and distended right abdomen were the common signs of cecal dilatation in cattle [12,17-20]. Sometimes, there are no visible clinical signs, and the dilated cecum is a coincidental finding on rectal palpation [17]. All vital parameters were usually normal in cattle with cecal dilatation, and hematological and blood biochemical analyses indices were not diagnostic but may be useful to estimate disease severity [3,21].

The intestinal disorders in bovine practice represented a large proportion of abdominal emergencies, and their definite diagnosis was a big challenge. This study aimed to describe different intestinal disorders in cattle in either the small intestine (SI) or large intestine (LI) with their sequelae, mainly peritonitis between loops of the SI, through establishing different ultrasonographic findings that were very characteristic to these affections.

## Materials and Methods

### Ethical approval

All animals were treated in accordance with guidelines established by the Animal Care and Use Committee at Faculty of Veterinary Medicine, Assiut University, Egypt, which basically conform to the Guide for the Care and Use of Laboratory Animals Care and Use of the National Institutes of Health in the USA (NIH publication No. 86-23, revised 1996).

### Study period and location

The study was conducted from October 2015 to May 2017 on cattle that subjected to treatment at the Veterinary Teaching Hospital, Faculty of Veterinary Medicine, Assiut University, Egypt.

### Animals

This study included 60 cattle. These animals included 20 clinically healthy non-pregnant cows (control group) and 40 diseased animals showing reduced appetite, distended abdomen, especially in the right side, and abdominal pain (diseased group). The diseased group included native (n=25) and Friesian (n=15) breed while the control group included native (n=12) and Friesian (n=8) breed. The control group consists of (n=20) 14 animals (between 4 and 6 years old) and six heifers (between 1 and 2 years old). The diseased animals (n=40) were between 6 months and 8 years. The control group included healthy non-pregnant cows (n=20) while the diseased group included males (n=15) and females (n=25). All diseased animals were suspected of having various intestinal disorders. Therefore, a complete case history was taken, and the animals underwent further investigations as follows:

### Clinical examination

Clinical examination of the animals was conducted using a clinical chart according to Cockcroft [22].

### Samples

A whole blood sample was collected on ethylenediaminetetraacetic acid and stored at 4°C until analysis. Blood serum samples were collected on plain vacutainer tubes and stored at −20°C until anal­ysis, according to Coles [23].

### Complete blood count

Various hematological indices were measured by a fully automated blood cell counter machine (Medonic CA620 Vet Hematology Analyzer, Stockholm, Sweden). The differential leukocytic count was determined using the four field meander method [23].

### Biochemical assays

The Spectro ultraviolet-Vis RS spectrophotometer (Labomed, Inc., Los Angeles, CA, USA) was used to determine serum concentrations of liver enzymes: Aspartate aminotransferase (AST), g-glutamyl transferase (GGT), and alkaline phosphatase, total serum protein, serum albumin, cholesterol, and triglycerides. Serum globulin was determined by subtraction of albumin from total protein, and its value was used to calculate the albumin/globulin ratio. Moreover, all kits and reagents were obtained from Gamma Trade Company, Cairo, Egypt.

### Ultrasonographic examination

The ultrasonographic examination was performed on standing, non-sedated animals after the application of transmission gel. Clipping of the hair was done from the area where the transducer was applied. The remaining hair was removed with a razor for optimal transmission of ultrasound waves. Ultrasonographic examination was conducted according to the previous studies [24-28] in diseased and healthy animals using a 3.5-MHz sector transducer connected with ultrasound apparatus (FF Sonic, Model UF-4000, Tokyo, Japan). The right flank and the last three right intercostal spaces (ICSs) were dorsally and ventrally scanned to determine different intestinal disorders.

### Statistical analysis

Data were analyzed using SPSS statistical software program for Windows, version 10.0.1 (SPSS Inc., Chicago, IL., USA). The obtained data were described as mean ± SD. The analysis of variance of the obtained data was performed using one-way analysis of variance, and the significance level was set at p≤0.05. The significant differences between the means of the control and diseased groups were evaluated.

## Results

### History and clinical findings

**Figure-1:**
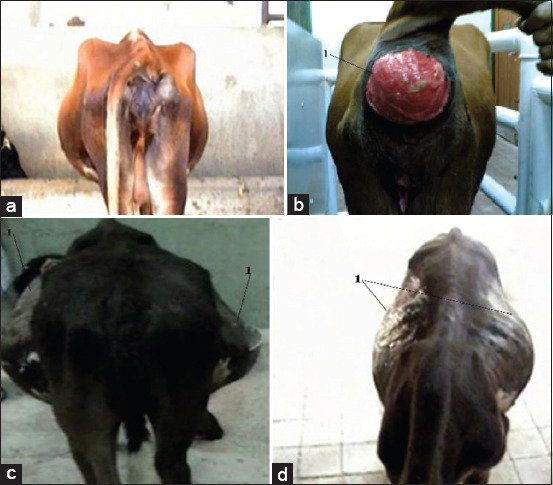
A 6-year-old-non-pregnant cow with proximal and partial ileus. It showed straining with rectal prolapse. 1: Prolapsed rectum (Figure-1a and b). Fattening bull 2 years with distal and complete intestinal obstruction showing severe symmetrical bilateral distension of the ventral abdomen with staggering gait. 1: Severe symmetrical bilateral distension of the ventral abdomen (Figure-1c and d).

**Figure-2:**
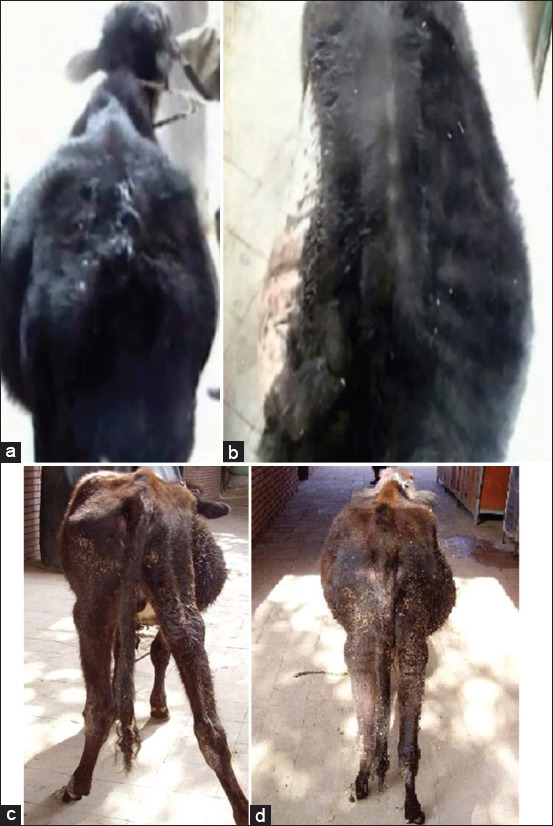
A 2-year-old Fattening bull with partial proximal ileus (intussusception). The animal showed normal body configuration (Figures-1b and 2a). Fattening bull 2 years with partial distal ileus. The animal showed slight right ventral distension of the abdomen (Figure-1d and 2c).

The most common clinical signs included reduced appetite, distended abdomen, especially in the right side, abdominal pain sensation on palpation, and tense abdomen. Of the 40 diseased animals, 30 animals expressed colic pain associated with constipation and straining. Rectal examination indicated an empty rectum with mucus and dilated SI loop, or dilated cecum loop, and/or colon. There was no alteration in body temperature, heart rate, and respiration. However, reduction (n=25) or absence (n=15) of ruminal motility was observed. In cases of complete IO, the animals showed elevated body temperature, heart and respiratory rates, mucous membrane congestion, including conjunctiva with engorged episcleral capillaries, and abnormal gait ataxia with movement reluctant with a tendency of recumbency (Figures-1 and 2).

### Blood picture

The diseased cattle either with ileus of SI or with cecal/colonal dilatation showed no significant changes in the whole blood picture. All blood indices within the reference ranges except for monocytic leukocytosis (Table-1).

**Table-1:** Mean values±standard deviation of blood picture and serum biochemical indices in the control and diseased groups of cattle.

Parameters	Control n=20	Ileus of SI n=30	Caecal/colonal dilatation n=10	Reference values
RBCs (×10^12^/L)	7.56±1.42	6.86±1.76	9.08±1.48	5-10 [33,34]
PCV (%)	34.62±5.84	36.20±6.56	3.26±4.88	24-46 [33,34]
Hb (g/L)	112.6±12.30	109.1±26.11	120.62±34.24	80-150 [33,34]
T.WBCs (×10^9^/L)	8.24±2.86	16.02±3.63*	18.52±4.77*	4-12 [33,34]
Neutrophiles (%)	32.46±5.22	28.78±4.82	30.26±6.05	26.56±4.13 [1,7]
Lymphocytes (%)	56.28±4.68	53.70±6.2	45.3±3.88*	62.38±3.23 [1,7]
Monocytes (%)	7.75±2.63	15.77±4.68*	17.02±4.92*	6.75±2.63 [1,7]
Eosinophiles (%)	4.31±1.56	5.11±1.25	6.12±1.00	3.31±0.07 [1,7]
Band cells (%)	1.20±0.30	1±0.2	1.3±0.66	1.40±0.52 [1,7]
Total proteins (g/L)	82.65±6.23	62.10±7.81*	58.08±4.93*	57-81 [33,34]
Albumin (g/L)	48.44±4.08	30.46±2.16*	28.33±2.06*	30-40 [32]
Globulin (g/L)	34.21±2.76	31.64±5.14	29.75±4.33	37.8±0.20 [33]
GGT (U/L)	12.68±5.10	30.2±5.49*	15.98±3.77	6.1-17.4 [33,34]
ALK (U/L)	36.36±5.26	42.97±9.32	65.51±4.88*	36.36±5.26 [1,7]
AST (U/L)	42.37±6.17	48.99±8.05	90.64±12.38*	78-132 [33,34]
Cholesterol (mmol/L)	9.93±1.26	12.03±2.60	10.66±1.87	6.24-22.13 [33]
Triglycerides (mmol/L)	5.11±1.25	6.09±2.95	4.87±1.08	0.113±0.09 [31]

RBCs=Red blood corpuscles, PCV=Packed cell volume, Hb=Haemoglobin concentration, TWBCs=Total white blood cells count, AST=Aspartate aminotransferase, ALK=Alkaline phosphatase, GGT=γ-Glutamyl transferase.

* Significant the values at ileus of SI group or that of caecal dilatation group compared with those at control group (*p<0.05). Reference values according to Khalphallah *et al*. [1], Khalphallah *et al*. [7], Morrow *et al*. [31], Rosenberger [32], Radostits *et al*. [33], Jackson and Cockcroft [34]

### Serum biochemical findings

Total proteins and albumins were significantly (p<0.05) reduced in both SI ileus and cecal/colonal dilatation when compared with the control group (Table-1). Animals with SI ileus had a remarkable (p<0.05) elevation in serum GGT concentrations. Meanwhile, there were no significant changes in serum levels of ALK, AST, cholesterol, and triglycerides when their values were compared with those of the control group (Table-1). Animals with cecal/colonal dilatation showed a remarkable (p<0.05) increase in serum ALK and AST concentrations. Meanwhile, no significant changes in serum GGT levels, cholesterol, and triglycerides were reported when their values were compared with those of the control group (Table-1).

### Ultrasonographic findings

The intestinal tract in the control group was imaged from the right flank region and ventral part of the last right three ICSs. The descending part of the duodenum had an echogenic envelope with a diameter of 1.8-4.2 cm (3.2±1.6 cm). It was imaged from the dorsal part of the right flank region and medially situated adjacent to the right abdominal wall. The jejunum and ileum were imaged as loops with two echogenic walls in cross-section with echoic or hypoechoic contents and a diameter of 2.7-4 cm (2.65±0.81 cm). Moreover, the normal peristaltic movement of the SI was imaged.

The cecum, proximal colon loop, and spiral colon could be imaged from the right flank region. The closest wall of the proximal colon loop and cecum was imaged as a continuous or slightly curved echogenic line, while their furthest wall was not imaged. The closest wall of the spiral colon was imaged as a garland-like appearance with small arched echoic lines attached next to each other. However, the furthest wall of the cecum and colon could not be imaged. Many intestinal disorders were diagnosed by ultrasonography in the diseased group. These disorders were either restricted to the intestine or extended to outside the intestinal tract. Table-2 summarizes the most common ultrasonographic findings of the different intestinal disorders in cattle.

**Table-2:** Differential diagnosis of intussusception, proximal and distal ileus obstruction (partial/complete) and caecal and/or colonal dilatation in cattle.

Parameters	Intussusception n=3	Proximal ileus with obstruction		Distal ileus with obstruction		Caecal/colonal dilatation (n=10)
	
		Partial (n=5)	Complete (n=7)	Partial (n=6)	Complete (n=9)	

Site of the probe						

The right flank and the last right 3 ICSs						
**Relationship to other organs**						
1. Duodenum	Duodenum intussusception	Dilated	Dilated	Imaged (not dilated)	Not imaged and sometimes imaged (Prestenotic dilated loops)	Not imaged
2. Jejunum and ileum	Imaging of some normal loops of SI	Imaging of some normal loops of SI	Not imaged	Imaged (Dilated loops)	Imaged (Dilated prestenotic loops and empty poststenotic loops)	Not imaged
3. Large intestine	Not imaged	Imaged normal	Not imaged	Imaged	Not imaged	Imaged
4. Liver	Imaged Affections were intertangled with liver dorsally and occupy the ventral part of the last right 3 ICSs			Imaged (The dilated loops sometimes interfered with the liver in the dorsal part of the last R- 3 ICSs)	Imaged Dilated loops duodenum filled the ventral parts of the last right three intercostals spaces and intertangled with the liver dorsally	Imaged Intertangled with the dilated caecum or colon
5. Right kidney	Not imaged	Imaged	Not imaged	Imaged	Not imaged	Not imaged
**Ultrasonogram**						
1. Image description	Multiple concentric rings	Dilated loops of the small intestine filled the whole right flank region, the ventral parts of the last right three intercostals spaces and intertangled with the liver in the dorsal part of the last right three intercostals spaces.				Imaged immediately adjacent to the right flank region with thick semi-circular echogenic wall (closest wall). Dilated caecum and colon may extend and make invisualization of loops of small intestine in the right flank region and last right three intercostals spaces while they intertangled with the liver the last two intercostals spaces.
2. Number and diameter of dilated loops	One (Bull eye´s lesion) loop 6.5-9.9 cm (8 cm)	One loop or less than 5 loops > 8 cm (6.5-9.9 cm)	One loop or less than 5 loops >15 cm (17-20 cm)	Multiple loops (More than 5 loops) <5.8 cm (4.4-6.8 cm)	Multiple loops (More than 5 loops) <5.8 cm (4.4-6.8 cm)	0
**Peristaltic movement of SI**						
	Slightly Reduced	Slightly Reduced	Completely reduced (ceased)	Slightly Reduced	Completely reduced (ceased)	Completely reduced

SI=Small intestine

**Figure-3:**
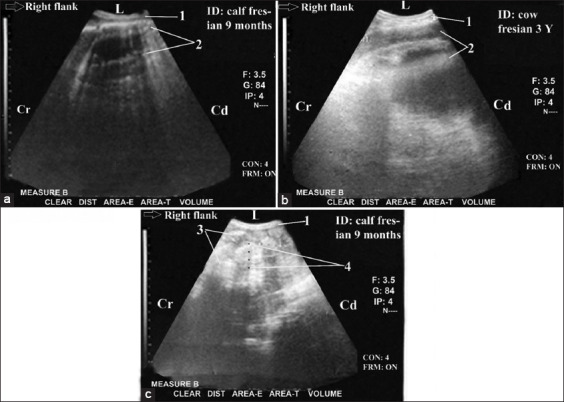
Ultrasonogram in a 9-month-old-Fresian male calf or a 3-year-old-non-pregnant cow with partial proximal ileus (intussusception of the duodenum) that was imaged from the dorsal and middle right flank region. It showed bull’s eyes lesion (partial and proximal ileus). The bull’s eyes lesions were visualized as two concentric rings with outer echogenic wall and hypoechoic lumen then inner highly reflective rings with anechoic center (Figure-3a and b). Visualization of normal loops of small intestine in some images (Figure 3c). 1: Abdominal wall. 2: Intussusception of the duodenum (bull^´^s eyes lesions). 3: Normal loops of SI. 4: diameter=2.67 cm. L: lateral. Cr: cranial. Cd: caudal.

Cattle with SI obstruction (n=30) showed a reduction in the peristaltic SI movement (either complete or incomplete). The diameter of the dilated duodenum was 6.5-9.9 cm (8.2±1.9 cm), and the diameter of the dilated jejunum or ileum was 4.4-6.8 cm (5.8±1.4 cm). Duodenum intussusception (n=3) was diagnosed as a form of partial proximal ileus. It was imaged from the dorsal right flank region and the right 11^th^ and 12^th^ ICSs in cross-section as bull’s eyes (bowel within bowel) lesions. The bull’s eye lesions were visualized as two concentric rings with an outer echogenic wall and hypoechoic lumen with inner highly reflective rings with anechoic center (Figure-3a-c). Some ileum and jejunum loops were imaged in case of duodenum intussusception. Hence, the right kidney and LI were not imaged. In the case of partial proximal ileus (n=5), the ileus site was more proximal in the duodenum, and the diameter of its dilated loop was 7-9 cm with slightly reduced peristaltic movement (not ceased). The dilated loop interfered with liver lobes. It obscured some parts of the liver in the dorsal and ventral right 12^th^ ICS and extended cranially to intertangle with the cranial border of the liver in the right dorsal and ventral 10^th^ ICS.

**Figure-4:**
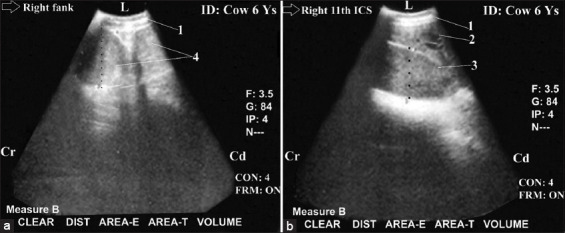
Ultrasonogram in a 6-year-old–non-pregnant female cow with partial and distal ileus imaged from the right flank region (Figure-4a) and from of the right 11^th^ intercostal spaces (ICS) (Figure-5b). It showed CS in several dilated loops of jejunum and ileum (=6.29 cm) as they had two echogenic walls with hypoechoic contents (Figure-4a). The peristaltic movement of SI was reduced but was not completely ceased. The duodenum (cranial duodenum=3.9 cm) was imaged normally (Figure-4b) from the right 11^th^ ICSs, and in close relation to the liver which was normally visualized from the dorsal part of this ICS. Cranial duodenum was appeared in CS as loop as loop with two echogenic walls and relatively echoic contents. 1: Abdominal wall. 2: Dilated loops of jejunum and ileum (=6.29 cm). 3: Cranial duodenum =3.9 cm. 4: Liver. L: lateral. Cr: cranial. Cd: caudal.

Cattle with partial distal ileus (partial distal obstruction; n=6) showed several dilated jejunum and ileum loops (n>5) through the right flank region with a diameter ranging between 4.4 and 6.8 cm. The peristaltic SI movement was slightly reduced but was not completely ceased. The duodenum (cranial duodenum), liver, proximal cecum loop, and/or colon and right kidney were visualized (Figures-4a and b).

**Figure-5:**
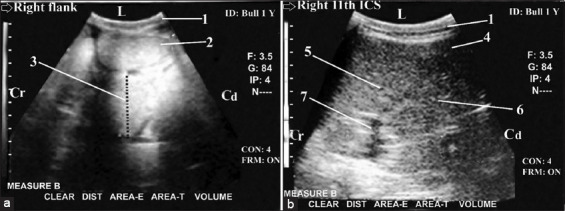
Ultrasonogram in a1-year-old-fattening bull with severe intestinal obstruction (Distal ileus with complete obstruction) (Figure-5a) and severe liver cirrhosis (Figure-5b). It was imaged from the right flank region (Figure-5a) and from the right 11^th^ intercostal spaces (Figure-5b). It showed cross-section in dilated loops of the small intestine (N>5 loops and diameter <6 cm [5.60]) with paralytic ileus and imaging of their two hyperechoic walls with echoic contents. Liver cirrhosis was imaged as multiple hyperechogenic foci within the hepatic parenchyma with anechoic nodules, reduced lumen of caudal vena cava (CVC) and heterogenic nature of hepatic parenchyma. 1: Abdominal wall. 2: Dilated loops of SI. 3: Diameter=5.60 cm. 4: Liver. 5: Anechoic nodules. 6: hyperechoic foci.7: Reduced lumen of CVC. L: Lateral. Cr: Cranial. Cd: Caudal.

**Figure-6:**
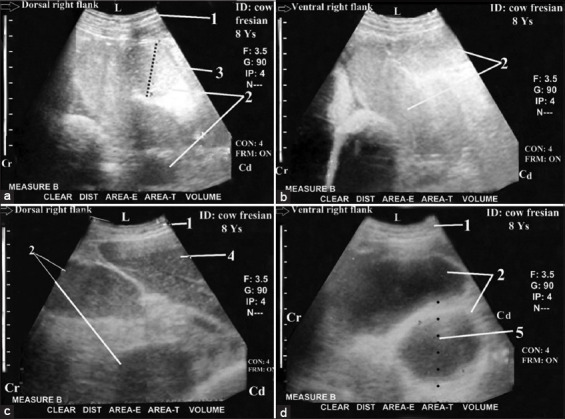
Ultrasonogram in an 8-year-old-non-pregnant cow with severe intestinal obstruction with paralytic ileus (distal ileus with complete obstruction). It was visualized from the right flank region (Figure-6a: dorsal and 6b: ventral) and from the right 12^th^ intercostal spaces (ICS) (Figure-6c: dorsal and 6d: ventral) showed cross section in the dilated loops of jejunum and ileum (N >5 loops and diameter <6 cm [5 and 5.3]) with imaging of their two wall (The closest and the furthest) that occupied most of the dorsoventral parts of the right last three ICSs (Figure-6c and d), intertangled with the liver (Figure-6c) and filled the whole right flank region with in visualization of the large intestine and right kidney (Figure-6a and b). 1: Abdominal wall. 2: Dilated loops of jejunum and ileum. 3: Diameter=5 cm. 4: Liver. 5: 3: Diameter=5.3 cm. L: Lateral. Md: Medial. Cr: Cranial. Cd: Caudal.

Ultrasonography was helpful in the diagnosis of distal ileus with complete IO (complete distal ileus; n = 9; Figure-5a). IO was associated with liver cirrhosis in a few cases (n=3; Figure-5b). The obstruction site was more distal because the number of dilated loops was more than five loops and the diameters ranged between 4.5 and 7 cm. A complete reduction of the peristaltic SI movement was observed. These dilated loops completely occupied the right flank region with complete obscuring (non-visualization) of both the LI (colon and cecum) and the right kidney (Figures-6a and b). They also occupied the last three right ICSs ventrally and intertangled with the liver dorsally (Figures-6c and d). Visualization of liver cirrhosis was imaged from the right flank just caudal to the last and exhibited multiple heterogenic echogenic areas in the hepatic parenchyma with less distinct imaging of the hepatic and portal structures (Figure-5b).

**Figure-7:**
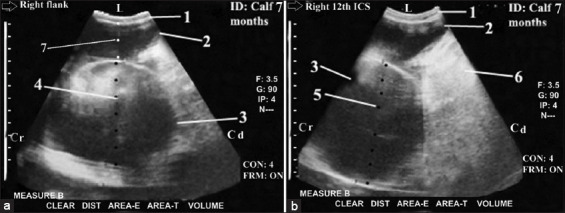
Ultrasonogram in a 7-month-old-calf with intestinal obstruction, paralytic ileus (Proximal ileus with complete obstruction) and liver fibrosis. It was visualized from the right flank (Figure-7a) and from the right l2^th^ intercostal spaces (ICS) (Figure-7b). It showed CS in dilated loop of the duodenum (N=1 or <5 loops and diameter >15 cm [17-20 cm]) that occupied the whole right flank region and the ventral parts of the right last three ICSs with in visualization of the loops of jejunum and ileum, large intestine, and right kidney (Figure-7a). Liver fibrosis was imaged as hyperechoic foci within the hepatic parenchyma with its characteristic heterogeneous nature (b). 1: Abdominal wall. 2: Dilated cranial duodenum. 3: Dilated descending duodenum. 4: Diameter = 16.1 cm. 5: Diameter = 16.43 cm. 6: Liver fibrosis. 7: Diameter= 6.8 cm. L: Lateral. Cr: Cranial. Cd: Caudal.

**Figure-8:**
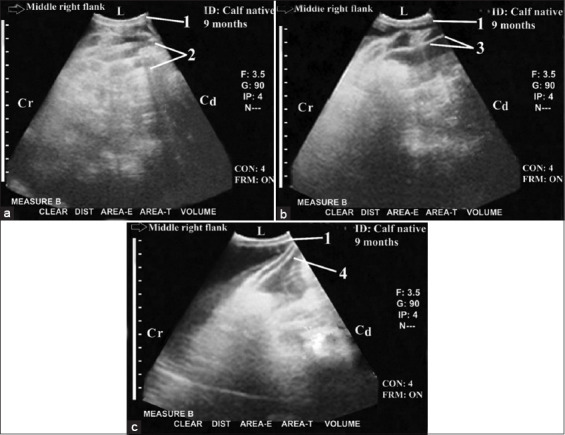
Ultrasonogram in a 9-month-old-native calf with peritonitis in the right flank. It was imaged from the middle part of the right flank region. It showed net-work of echogenic fibrinous deposits between loops of the small intestine (SI) led to adhesions and in visualization of SI. These fibrinous deposits interspersed with hypoechoic fluids forming network of echogenic fibrinous deposits which appeared as fibrinous echogenic honey-comb shaped cells (Figure-8a), spider-web type appearance (Figure-8b) or fibrinous echogenic bands (Figure-8c). 1: Abdominal wall. 2: Honey-comb shaped cells. 3: Spider-web type appearance. 4: Fibrinous echogenic bands. L: Lateral. Cr: Cranial. Cd: Caudal.

Complete and proximal IO in the duodenum area (complete proximal ileus) were seen (n=7; Figures-7a and b) with peritonitis (n=3; Figure-8). The obstruction site was more proximal at the duodenum area because the dilated loops were less than five loops (one loop), and its diameter ranged between 15 cm and 20 cm. Complete reduction of the peristaltic SI movement was reported with non-visualization of the jejunum, ileum, LI (colon and cecum), and right kidney. The dilated loops occupied the last three right ICSs ventrally and intertangled with the liver dorsally.

**Figure-9:**
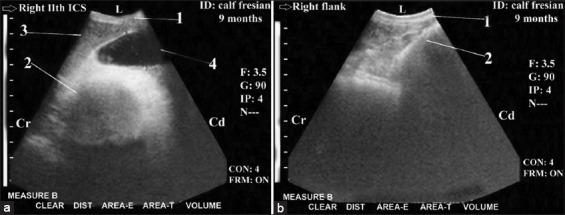
Ultrasonogram in a 9-month-old-fresian calf with cecal dilatation. It was imaged from the right 11^th^ intercostal spaces (ICS) (Figure-9a) and from the right flank (Figure-9b). It showed C.S. in the dilated loops of the closest wall of the caecum and/or colon that imaged as thick semi-circular echogenic line immediately adjacent to the right abdominal wall in the right flank region and in the last three intercostal spaces. The dilated loop was intertangling with the liver in the dorsal part of the last right three intercostal ICSs (Figure-9a) and obscuring small intestine and right kidney (Figure-9b) in the right flank region. 1: Abdominal wall. 2: Dilated caecum and/or colon. 3: Liver. 4: Gall bladder. L: Lateral. Cr: Cranial. Cd: Caudal.

Peritonitis (n=3) was imaged as a network of fibrinous echogenic deposits between the SI loops with interspersing hypoechoic fluids (honeycomb-shaped cells; Figure-8a). Peritonitis was also imaged as spider web-type appearance (Figure-8b) or fibrinous echogenic bands (Figure-8c). Peritonitis caused SI loop adhesions, complete intestinal peristaltic movement reduction, and non-visualization of both SI and LI. Ultrasonography could diagnose cecal dilatation (n=10) in which the SI was not imaged with complete peristaltic movement reduction (Figure-9). The dilated loops of the cecum and colon were immediately imaged adjacent to the right abdominal wall. The closest wall of the cecum appeared as a thick semicircular echogenic line that was medially located to the right flank region. The dilated cecum and colon caused non-visualization of SI loops in the right flank region and in the last three ICSs ventrally while they intertangled with the liver at the last two right ICSs. They also obscured the right kidney.

## Discussion

Gastrointestinal disorders are common in bovine, and recent advances in their differential diagnosis are recorded [29]. Therefore, the present study used ultrasonography to differentiate these disorders with successful results. The recorded case history and clinical findings of the intestinal disorders in this study are similar to those reported in the previous studies [5,8,12,15,19]. Rectal examination findings (e.g., dilated SI loops, absence of feces in the rectum or feces containing blood, mucus, or fibrin) help to diagnose intestinal disorders in cattle. This is in agreement with the findings of earlier works [19]. Moreover, the current study recorded that rectal palpation is a more important diagnostic tool for cecal dilatation than swinging and/or percussion auscultation. Similar findings were reported in the previous studies [5,8,18,19]. Although the clinical signs are valuable for the preliminary diagnosis of intestinal disorders in cattle, definite diagnosis is based on other investigations (e.g., laboratory and ultrasonography findings) [5,8].

Monocytic leukocytosis in this study was recorded in the SI ileus and cecal/colonal dilatation. These findings follow those mentioned by the findings of other authors [8,11,15,30]. The other blood picture indices in the present study were within the reference values reported by Khalphallah *et al*. [1], Morrow *et al*. [31], Rosenberger [32], Radostits *et al*. [33], and Jackson and Cockcroft [34]. In general, speaking, the hematological values are normal in most of the recorded intestinal disorders unless cecum necrosis accompanied by peritonitis exists [19,21].

Hypoproteinemia and hypoalbuminemia were observed either in the SI ileus or in cecal/colonal dilatation. Khalphallah *et al*. [5,8] reported significant hypoproteinemia and an increase in serum AST and ALK activities in cases of IO and cecal dilatation in buffaloes. Moreover, Maclachlan and Cullen [35] reported elevated serum concentrations of total proteins and globulins in the intestinal disturbances in large ruminants. Animals with SI ileus showed a remarkable elevation in serum GGT concentrations. Meanwhile, animals with cecal/colonal dilatation showed a remarkable increase in serum ALK and AST concentrations. These findings relatively agreed with the findings of a previous study [36]. The current results showed no significant changes in blood concentrations of cholesterol and triglycerides. The same reports were mentioned by Khalphallah *et al*. [8] in the case of cecal and/or colonic dilatation. In contrast, Rosenberger [32] stated that the total serum cholesterol level fell during acute inflammatory degenerative disease and enteritis.

Ultrasonography had a very important diagnostic and prognostic significance in evaluating different intestinal diseases in cows and buffaloes [5,8,37]. Therefore, the present study depended on ultrasonography for differential diagnosis of the recorded intestinal disorders. In the current work, the SI and LI ultrasonography in the control group was conducted to establish a reference image to compare with the diseased group. The intestinal tract imaging in healthy cattle could be successfully conducted from the right flank and the last three ICSs. The ultrasonography findings of normal SI and LI agreed with those reported by Braun and Marmier [25], Braun and Amrein [26] in cattle, and by Khalphallah *et al*. [6] in buffaloes.

Ultrasonography could detect the ileus site, either proximal or distal, by assessing the number and diameter of dilated loops. The ileus site is distal at the jejunum or ileum area when the number of dilated loops is more than five loops, and their diameter is <5.8 cm. If the number of the dilated loops is <5 with a diameter of >15 or >8 cm, the ileus site is more proximal in the duodenum area with either complete or partial obstruction, respectively. Similar findings were reported in cattle before [38]. Moreover, there was reduced peristaltic movement in the case of SI obstruction (complete or incomplete). This is in agreement with the results of a previous study in buffaloes [5].

Partial or complete IO was associated with reduction or absence of peristaltic SI movement, respectively. In both proximal and distal partial obstructions, the LI and right kidney could be visualized, while they could not be imaged in case of complete obstruction because the dilated SI loops fully occupied the right flank region and the ventral part of the last right three ICSs, and intertangled with liver lobes dorsally. These findings are relatively supported by Braun *et al*. [38]. Moreover, Khalphallah *et al*. [5] found empty poststenotic ileum and jejunum loops with anechoic contents in buffaloes with distal ileus.

Duodenum intussusception was also diagnosed in the present study as a form of partial proximal IO where it was imaged in cross-section as bull’s eyes lesions. Similar findings were recorded by Braun *et al*. [38] and Khalphallah *et al*. [5] in cattle and buffaloes, respectively. The intussusception site was the duodenum. This was due to the visualization of normal jejunum and ileum (empty loops) loops, and the number of the dilated loops was one. The right kidney and LI were not imaged, and reduction of intestinal motility was not observed. Khalphallah *et al*. [5] mentioned that buffaloes with intussusception had slightly reduced peristaltic SI movement with non-visualization of the right kidney and LI.

Interestingly, ultrasonography could diagnose different complicated intestinal disorders in the examined cattle, such as IO of jejunum or ileum (distal and complete) with liver cirrhosis or IO of the duodenum (proximal and complete) with peritonitis. The SI peristaltic movement in case of complicated intestinal cases usually ceased. The reported ultrasonography findings of liver cirrhosis were similar to those reported by Braun *et al*. [39] and Khalphallah *et al*. [7]. Hence, the hepatic parenchyma was of a heterogenous nature because it contained several heterogenic echogenic foci with reduced imaging of the hepatic and portal structures.

Peritonitis caused adhesions of SI loops with complete reduction of their peristaltic movement. Moreover, non-visualization of both SI and LI with interspersing hypoechoic fluids (spider web-type appearance) was also observed. Peritonitis between SI loops was also imaged as echogenic bands or honeycomb-like deposits with hypoechoic fluid accumulation that agreed with Braun *et al*. [40]. It was also imaged as echogenic fibrinous deposits with a honeycomb-like appearance and fluid accumulation. Moreover, similar findings were previously mentioned by Abu-Seida and Al-Abbadi [29].

Ultrasonography could diagnose cecal and/or colonal dilatation. The dilated loops of the cecum and/or proximal loop of the colon were imaged immediately adjacent to the right abdominal wall with complete non-visualization of the SI and right kidney and complete reduction of the peristaltic movements. Other reports mentioned that the closest wall of the dilated cecum or colon appeared as a thick semicircular echogenic line directly medially to the right flank region, the furthest wall, and contents of the cecum. Moreover, the proximal colon loop could not be imaged due to the gas reverberation artifacts [19,27]. Furthermore, the currently reported findings added that dilated cecum and/or colon extended to the last right three ICSs because they were intertangled with the liver dorsally and obscured SI loops ventrally. In addition, Khalphallah *et al*. [8] reported similarity in the ultrasonographic appearance of the cecum and proximal loop of the colon. Therefore, differentiating between them, either in healthy or diseased buffaloes, was difficult.

## Conclusion

The current study differentiated between different intestinal disorders in cows based on ultrasonographic findings and other traditional diagnostic methods. It diagnosed proximal SI ileus (either partial or complete) and distal SI ileus (either partial or complete). Cecal and/or colonal dilatation was also imaged ultrasonographically in the current study. Peritonitis or liver cirrhosis was diagnosed either as a complication of intestinal disorders or associative disorders in cattle. The limitations or technical difficulties implementing this methodology included the availability of an expert operator with thorough knowledge of ultrasonographic examination and ultrasound apparatus availability.

## Authors’ Contributions

AK, SAM, and EE: Prepared conception and design of study. AK, HKE, MAH, SAM, AMA, and EE: Conducted the field study, cattle examination and ultrasonographic examination. AK, DS, EE, HKE, and MAH: Collected laboratory samples and conducted biochemical analyses. AK, SAB, MAH, and DS: Performed analysis and interpretation of data. AK, DS, and HKE: Drafted the manuscript. AK, EE, SAM, SAB, and AMA: Carried out critical review and revision. All authors have read and approved the final manuscript.
